# Integration of geoscience frameworks into digital pathology analysis permits quantification of microarchitectural relationships in histological landscapes

**DOI:** 10.1038/s41598-020-74691-9

**Published:** 2020-10-16

**Authors:** Timothy J. Kendall, Catherine M. Duff, Andrew M. Thomson, John P. Iredale

**Affiliations:** 1 University of Edinburgh Centre for Inflammation Research, Queen’s Medical Research Institute, The University of Edinburgh, 47 Little France Crescent, Edinburgh, EH16 4TJ UK; 2 Edinburgh Pathology, The Royal Infirmary of Edinburgh, The University of Edinburgh, 51 Little France Crescent, Edinburgh, EH16 4SA UK; 3 Centre for Cardiovascular Sciences, Queen’s Medical Research Institute, The University of Edinburgh, 47 Little France Crescent, Edinburgh, EH16 4TJ UK; 4 NHS Lothian University Hospitals Division, Pathology Department, The Royal Infirmary of Edinburgh, 51 Little France Crescent, Edinburgh, EH16 4SA UK; 5 Senate House, University of Bristol, Tyndall Avenue, Bristol, BS8 1TH UK

**Keywords:** Biological techniques, Mechanisms of disease, Translational research, Ecology

## Abstract

Although gold-standard histological assessment is subjective it remains central to diagnosis and clinical trial protocols and is crucial for the evaluation of any preclinical disease model. Objectivity and reproducibility are enhanced by quantitative analysis of histological images but current methods require application-specific algorithm training and fail to extract understanding from the histological context of observable features. We reinterpret histopathological images as disease landscapes to describe a generalisable framework defining topographic relationships in tissue using geoscience approaches. The framework requires no user-dependent training to operate on all image datasets in a classifier-agnostic manner but is adaptable and scalable, able to quantify occult abnormalities, derive mechanistic insights, and define a new feature class for machine-learning diagnostic classification. We demonstrate application to inflammatory, fibrotic and neoplastic disease in multiple organs, including the detection and quantification of occult lobular enlargement in the liver secondary to hilar obstruction. We anticipate this approach will provide a robust class of histological data for trial stratification or endpoints, provide quantitative endorsement of experimental models of disease, and could be incorporated within advanced approaches to clinical diagnostic pathology.

## Introduction

Traditional diagnostic pathology remains the gold-standard means of assessing tissue but is a subjective and poorly reproducible craft. Progress has been made to introduce objectivity and reproducibility into the field by computational interrogation of digital histological images^1^ with direct and creative links to clinically actionable outcomes^2,3^. However, there remain both practical obstacles in the current approaches and opportunities for conceptual developments in a new and rapidly expanding field.

Current image analysis methods often require study-specific algorithm training by end-users. Such training impedes widespread adoption as it is time-consuming, and critically precludes inter-study comparison of measured outputs or outcomes in animal modelling of disease or clinical trials. Only by developing methods that can be extensively validated and applied uniformly and intuitively across studies without a need for specialist input can quantitative digital pathology disrupt classical subjective assessment in a research setting or within routine practice.

Further, current ‘black-box’ methods are unable to extract understanding from the histological context of observable features, the most critical component informing skilled subjective assessment, or provide histologically relatable insight that can be further utilised for mechanistic research. Feature recognition is central to both traditional and computational methods. Although advances in computational feature annotation using deep-learning methods^4–7^ have increased the accuracy of image segmentation, ‘real-world’ diagnostic acuity is a function of histological literacy—an appreciation of the histological context and relationships between features—rather than accurate feature recognition alone. Understanding from these feature relationships is not currently exploited computationally, representing a significant opportunity for a more creative approach to harness concepts with proven, real-world value.

We reasoned that any annotated histological image could be conceptualised as a simple two-dimensional landscape in a generalisable manner to permit quantitation of feature relationships by methods developed for landscape analysis in geosciences and ecology. We describe a generalisable scale-independent framework that leverages essential feature relationships using geoscience approaches. No further user-dependent training to operate on existing image datasets in a classifier-, species-, and disease-agnostic manner within computational workflows. This provides a pathologically intuitive framework that identifies occult abnormalities, derives mechanistic insights, and defines a new feature class for machine-learning disease classification.

## Results

### Fully classified histological images can be re-interpreted as categorical maps and analysed within a fully computational pipeline using landscape ecology and geosciences methodologies

**Figure 1:**
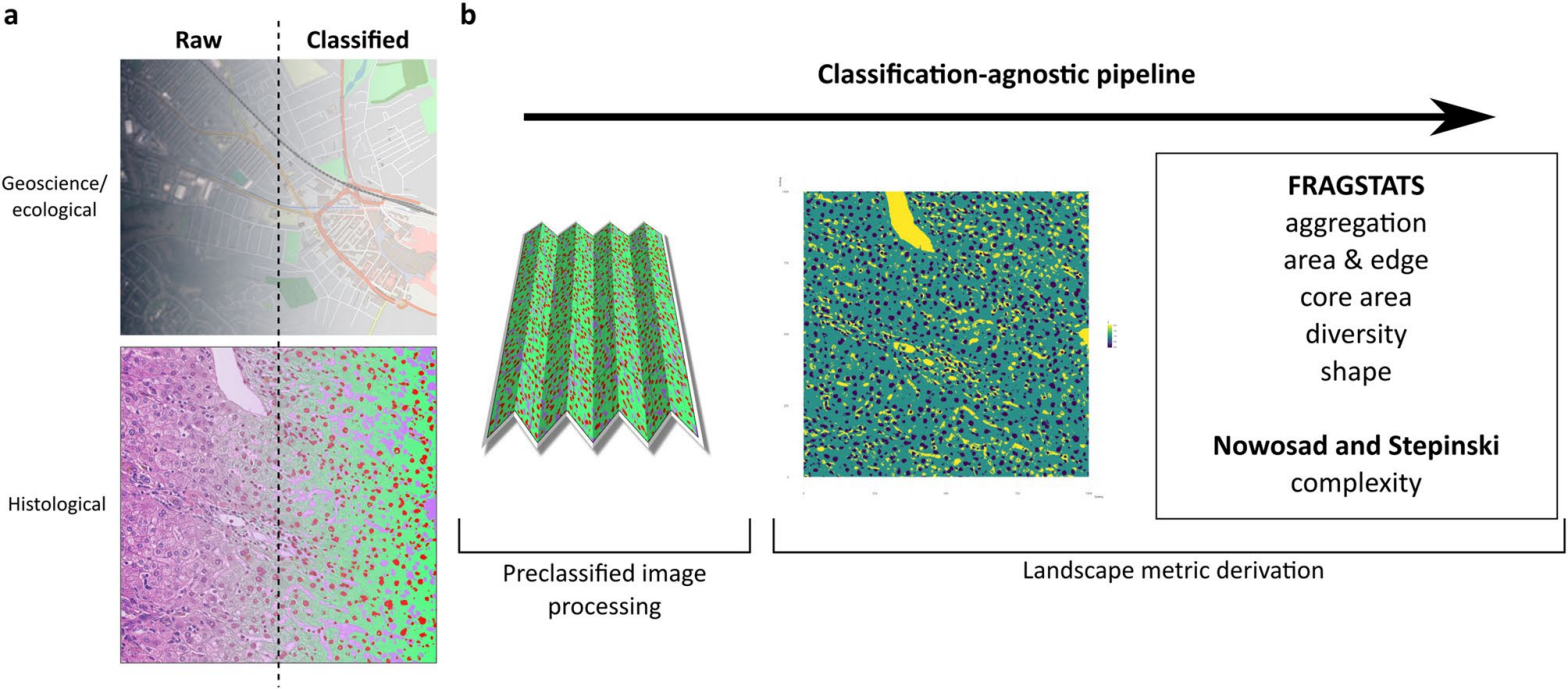
Fully classified histological images can be considered categorical maps and analysed as part of a fully computational pipeline using landscape ecology and geosciences methodologies. (**a**) Categorical representations of the landscape are routinely evaluated in landscape ecology and geosciences by specific tools. The generation of a fully segmented output image from a histological input, by any available method, is an analogous process differing only in scale (Contains modified Copernicus Sentinel data 2019 processed by Sentinel Hub under CC BY 4.0. Mapped tiles copyright OpenStreetMap contributors under Open Database Licence). (**b**) A pipeline using fully segmented images converts the images to an appropriate file format feeding the *landscapemetrics* package in R, generating the complete suite of metrics described in FRAGSTATS, and other holistic landscape measures of complexity and organisation.

The input for our analytic framework is a landscape pattern created by manual or computational annotation of a histological image. Complex computational methods to fully classify histological images are available, and their ease-of-use and accuracy continue to increase. The output of classifiers such as U-net^4^ can be a categorical landscape equivalent to those generated in large-scale mapping and geoscience studies. Whilst the scales differ, the fundamental nature of the data representation is the same (Fig. 1a).

In landscape ecology, categorical landscape patterns are mosaics of discrete areas (‘patches’) belonging to defined classes. Such patches are environmentally homogeneous areas with their boundaries reflecting the significant change in environmental conditions between them. Conceptually, the histological landscape also consists of a mosaic of ‘environmentally’ similar areas represented by tissues or cells and extracellular microarchitectural structures. Analysis of such categorical landscape patterns can generate metrics describing individual patches, the patch class, or defining the landscape as a whole. When applied to histological landscapes, class- and landscape-level metrics describe the topography of the tissue in a holistic and novel language whilst individual patch-level metrics provide metrics complementary to more traditional single-cell/group histological phenotyping^8^ provided by existing methods. We developed a pipeline using classified images to analyse the landscape patterns with methods derived from the FRAGSTATS suite^9^, a spatial pattern analysis program for categorical maps originally developed in association with the USDA Forest Service, as well as more recently described measures of landscape complexity^10^ (Fig. 1b).

**Figure 2:**
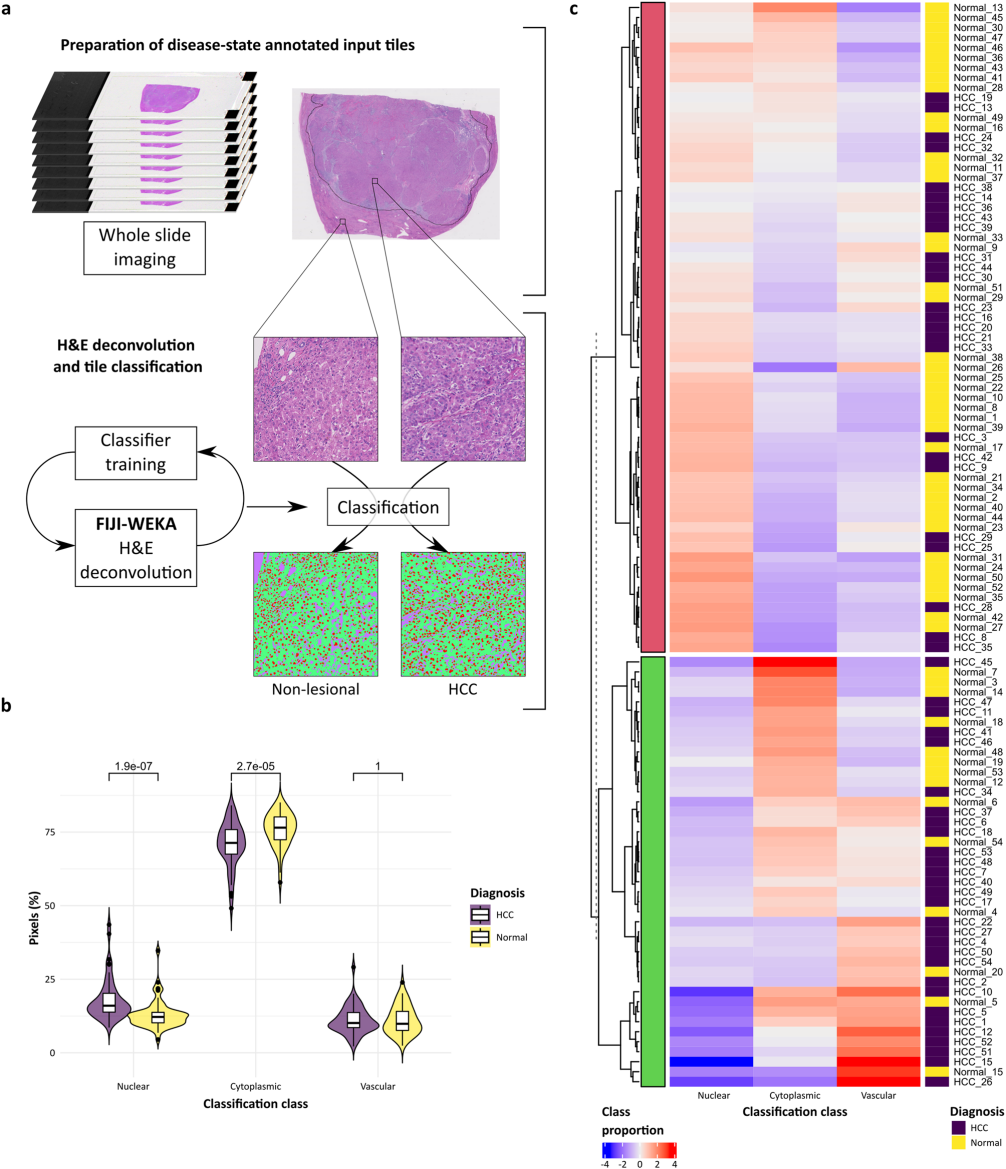
Standard pixel counts of classified image are limited value alone. (**a**) A dataset of 54 whole slide images of H&E-stained sections from liver resections for hepatocellular carcinoma were used as an exemplar for the added value derived by landscape analysis of classified images. Lesional (HCC) and non-lesional fields were selected from each slide and a simple WEKA machine-learning H&E deconvolution classifier trained to allow pixel classification of tiles into 3 classes (nuclear, cytoplasm, vascular channels). (**b**) Pixel-class proportions for ‘nuclear’ and ‘cytoplasmic’ were significantly different between HCC and non-lesional regions when analysed by group (violin plot with kernel density and median (centre line), first and third quartiles (lower and upper box limits), 1.5 × interquartile range (whiskers); p-values of unpaired two-sided two-sample t-test for each metric, n = 54). (**c**) K-means clustering using these three pixel-class proportions alone was poor at segregating regions by diagnosis.

As a first proof-of-principle, we used a set of 54 resection and explant liver H&E-stained slides containing primary liver cancer (hepatocellular carcinoma) and surrounding non-lesional liver. After whole-slide imaging we selected and tiled regions of lesional and non-lesional tissue and trained a basic machine-learning classifier using the WEKA plugin within FIJI, a readily available and commonly-used open-source tool^11,12^, to deconvolute the H&E staining into three simple classes—nuclei, cytoplasm and vascular channels (Fig. 2a). The output categorical image dataset was successfully employed within the pipeline in place of categorical earth sciences maps to generate the equivalent metrics.

### Landscape metrics provide a unique language for detailed histological phenotyping and represent an intuitive input dataset for machine-learning disease-classification methods

The most commonly used and simplest information available from a classified image is the number of pixels assigned to each class (Fig. 2b). In the classified liver dataset, the pixel-class proportions for lesional and non-lesional regions were significantly different on a group-wise basis but these three metrics alone did not provide good inter-group discrimination when used for unsupervised hierarchical k-means clustering (Fig. 2c).

**Figure 3:**
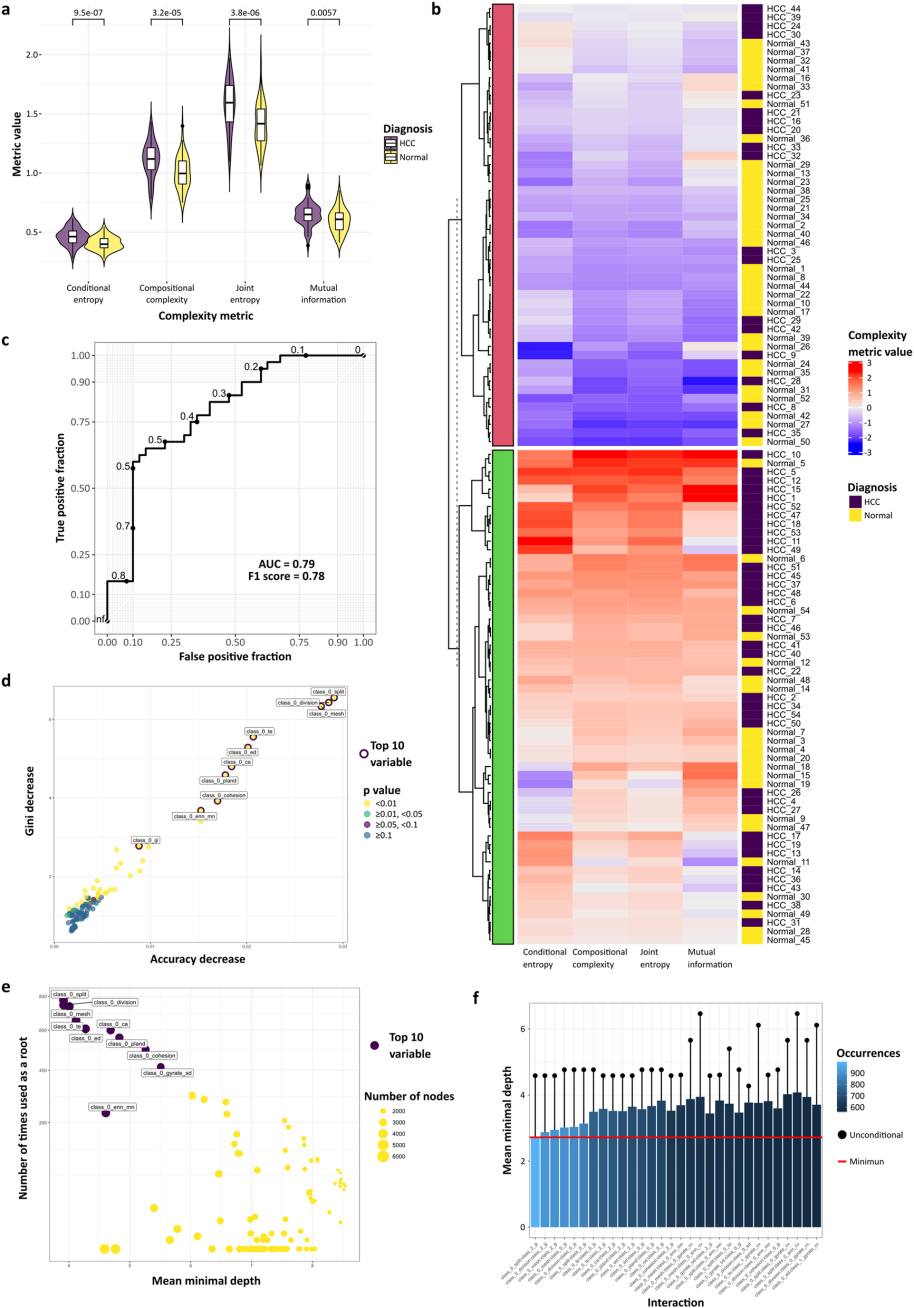
Classification-agnostic landscape metrics allow histopathological phenotyping in human disease and represent an intuitive input dataset for machine-learning disease-classification methods. (**a**) Individual complexity metrics from non-lesional liver and hepatocellular carcinoma can be used as discrete phenotyping measures (violin plot with kernel density and median (centre line), first and third quartiles (lower and upper box limits), 1.5 × interquartile range (whiskers); p-values of unpaired two-sided two-sample t-test for each metric, n = 54) or combined for improved segregation based on diagnosis after use in k-means clustering (**b**). (**c**) The complete suite of landscape metrics can be used for machine-learning diagnostic classification. For example, classification as normal or hepatocellular carcinoma using a simple random forest classifier was undertaken; Receiver Operating Characteristics curve with area under the curve (AUC) and F1 score for diagnostic accuracy on test set with thresholds marked (maximum F1 score at threshold value of 0.49). Examination of the variable importance in the constructed classifier reveals additional information about the importance of the features that can be translated back into subjective study; for example (**d**) Accuracy decrease (mean decrease of prediction accuracy after variable is permuted) versus Gini decrease (mean decrease in the Gini index of node impurity by splits on variable) with p-value of test determining whether the observed number of successes (number of nodes in which variable was used for splitting) exceeds the theoretical number of random successes, or (**e**) number of times used as a root (total number of trees in which variable is used for splitting the root node) versus mean minimal depth with number of nodes (total number of nodes that use variable for splitting). (**f**) Intuitive information about the pairwise variable importance was also available. Taken as a whole, interactions between nuclear features and those of the sinusoidal vasculature were computationally rated as the most important/frequent in distinguishing HCC from non-lesional tissue (class 0—nuclei, class 1—cytoplasm, class 2—vascular channels).

The four holistic metrics of landscape complexity^10^ from the *landscapemetrics* package provide single values derived from each landscape. These four metrics alone can be used as quantitative descriptors of the complete histological landscape to successfully define and quantify differences between paired tumour and normal liver, augmenting the subjective diagnosis (Fig. 3a). The four metrics in combination were used for unsupervised k-means clustering and provided improved disease discrimination compared with pixel-class proportions alone (Fig. 3b).

We reasoned that the larger suite of landscape metrics generated from categorical histological landscapes (Supplementary Table 1) could be used more effectively than simple pixel-class proportion alone in downstream applications such as machine-learning diagnostic classification. As a proof-of-concept, selected landscape- and class-level metrics from the same dataset were used as features for model training after randomly splitting cases into a training and test set. A random forest classifier was constructed from the selected features of the training set, and the predictive value of the model determined on the test set (Fig. 3c), demonstrating the applicability of this type of metric that can be generated entirely from a classified image in a pertinent down-stream use. Crucially, the landscape metrics are histologically meaningful and intuitive so that variable importance measures derived from the classifier construction provide additional value compared with alternative ‘black-box’ methods in current use with raw images. In our exemplar, the features derived from the ‘nuclear’ class in ‘aggregation’ and ‘area and edge’ categories are the most highly ranked in the classifier construction (Fig. 3d–f). These metrics represent nuclear morphology and distribution, critical features used by pathologists to make a subjective diagnosis, demonstrating that a fully computational landscape approach independently identifies and utilises features central to gold-standard traditional practice, and provides output in an intuitive and usable form. Simply, the landscape metric framework uses a common language with subjective observers that permits ready translation of insight from computational output back into human practice that alternative methods do not.

### Histological landscape patch analysis is classifier-, disease-, and tissue-agnostic

To demonstrate the classifier, disease, and tissue-type agnosticism of this landscape patch approach, whole-slide images of post-mortem thyroid in an alternative file format were downloaded from the GTEx Tissue Image Library. A set (n = 10) of H&E stained sections of thyroid regarded as normal or with the histological features of Hashimoto’s thyroiditis, an autoimmune disease characterised by lymphocytic inflammation and follicle destruction, by the reviewing pathologist were obtained. The whole-slide images can be used in the native file format by an alternative open-source bioimage application, QuPath, with internal down-scaling, or the whole-slide images can be down-scaled by extraction of the required resolution series using the Open Microscopy Environment’s Bio-Formats plugin^13^ within FIJI. This latter method was used to create smaller files, cropped more closely to the tissue, to allow quicker computation.

**Figure 4:**
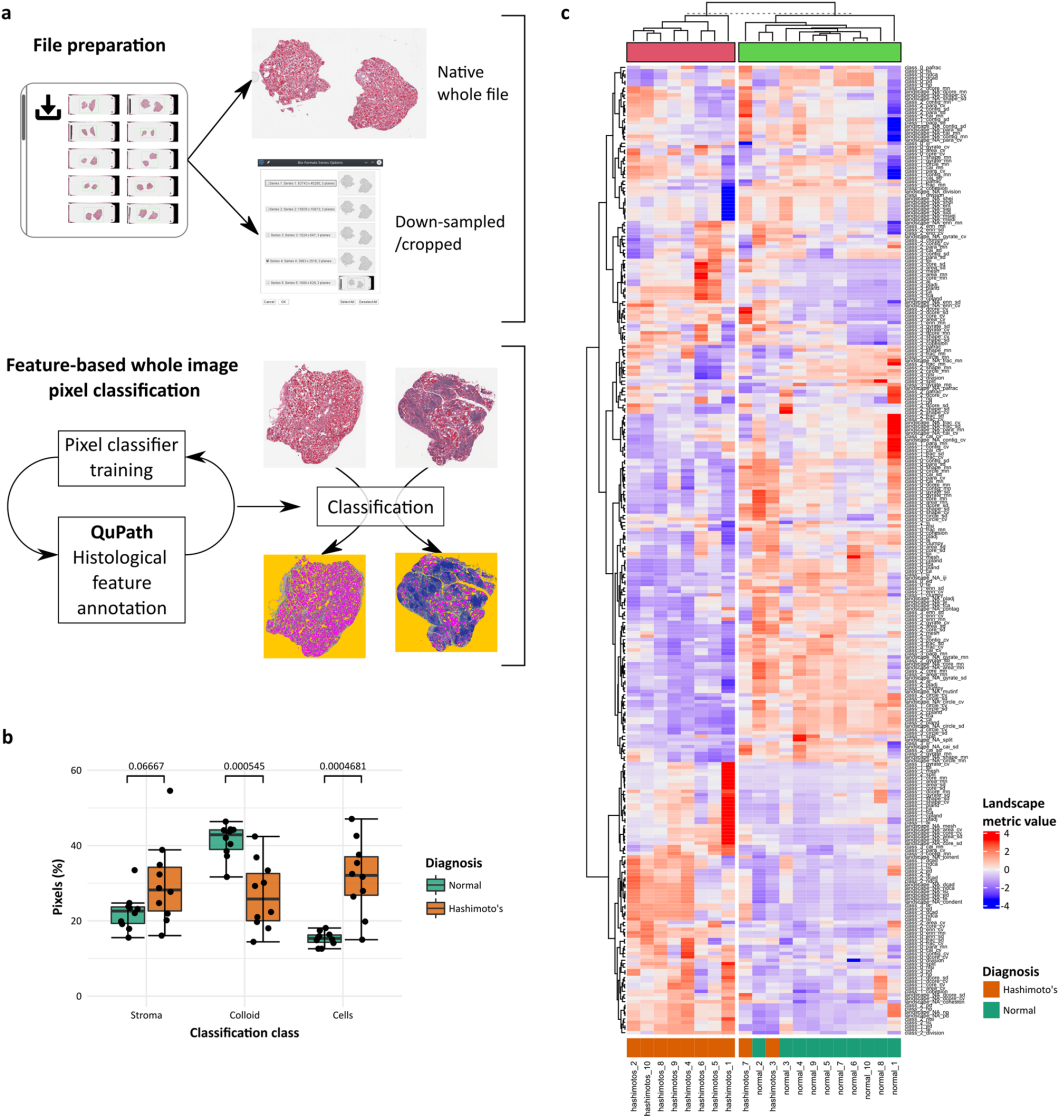
Landscape analysis can be applied to images from multiple organs with different diseases that have been classified by multiple methods and software. (**a**) A separate dataset of H&E stained sections of normal thyroid or thyroid showing Hashimoto’s thyroiditis (n = 10) from the GTEx Tissue Image Library were used to train a random trees classifier in QuPath after image down-scaling and cropping. Histological classes ‘cells’, ‘stroma’, ‘colloid’ and ‘space’ were used. (**b**) Histological pixel-class proportions for ‘cell’ and ‘colloid’ were significantly different between normal and diseased thyroid when analysed by group (individual points and median (centre line), first and third quartiles (lower and upper box limits), 1.5 × interquartile range (whiskers); p-values of Welch unpaired two-sided two-sample t-test for each metric, n = 10). (**c**) A full suite of landscape metrics derived from the classified images allowed segregation of cases effectively by k-means clustering.

A pixel-classifier of random trees (‘RTrees’) type with ‘gaussian’ and ‘weighted deviation’ features selected was trained within QuPath to classify pixels into the histological classes ‘cells’, ‘stroma’, ‘colloid’, and tissue-free space rather than tinctorial H&E deconvolution (Fig. 4a). Simple histological class-based pixel quantification of classified images demonstrated differences between normal and diseased thyroid (Fig. 4b), as would be expected in a disease characterised by inflammation. The QuPath-classified images could also be further incorporated into the landscape patch pipeline in the same manner as WEKA-classified tiles to generate a full suite of landscape metrics that provided good disease discrimination of individual cases (Fig. 4c).

### Spatial point pattern analysis of discrete features complements landscape patch analysis of classified images and provides quantitative support for subjective evaluation

A classified categorical image can not only be used within a landscape patch pipeline but can also be used to generate spatial point patterns. Such point patterns allow interrogation of histological features using an alternative framework from the ecological sciences that evaluate relationships between features that subjective histological assessment often relies upon. Further, point pattern analysis is complementary to landscape patch analysis and can be undertaken on the same images. A spatial point pattern of marked features in 2-dimensional space allows simple measures of feature density and distance to be calculated, and the clustering and dispersal of annotated features can be quantified by well-characterised specialised mathematical functions^14^.

**Figure 5:**
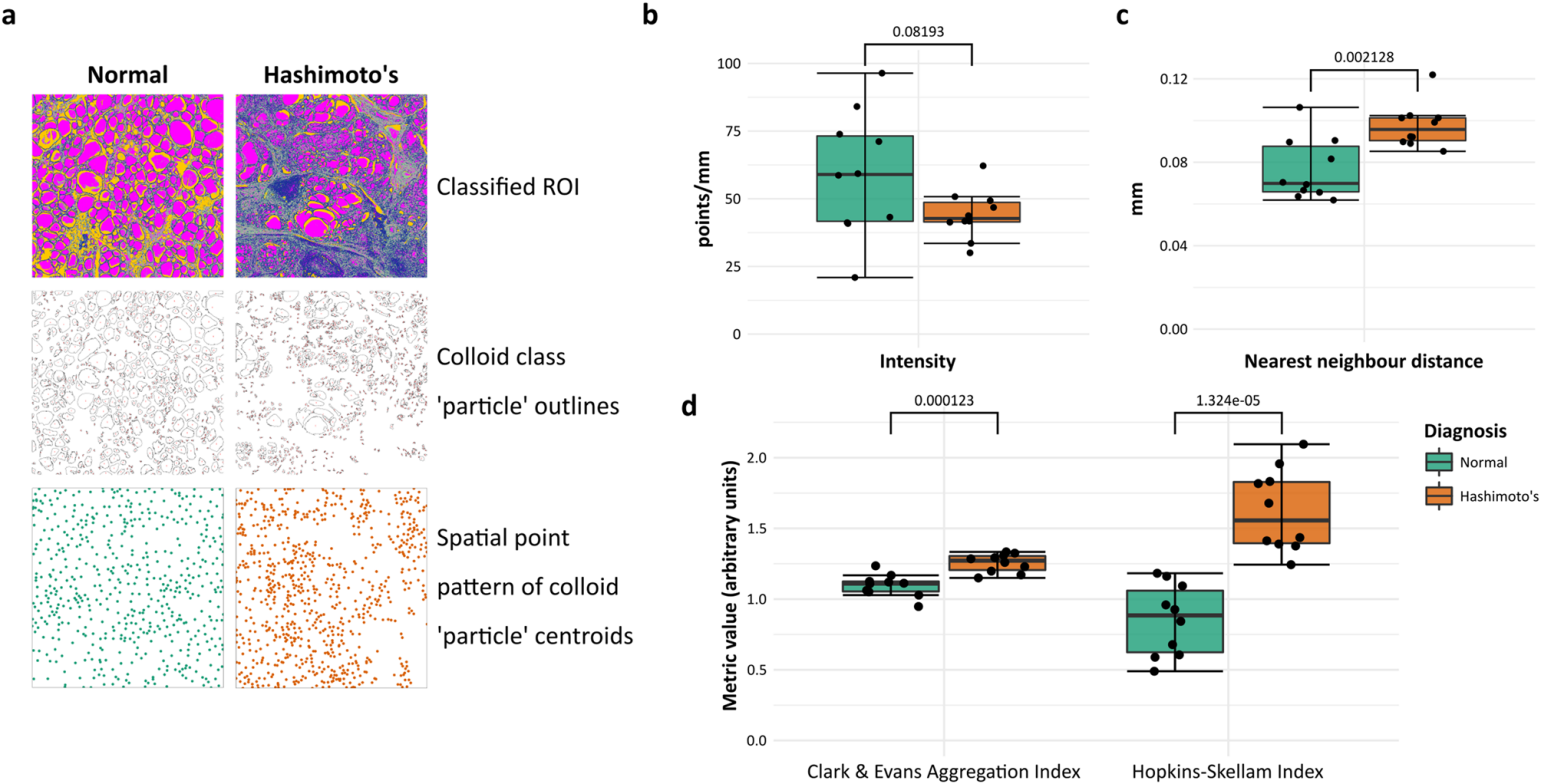
The relationships and organisation of histological features in a classified image can be interrogated through generated spatial point patterns. (**a**) Uniform windows from the classified thyroid image dataset were used to generate spatial point patterns of the centroids of the ‘colloid’ class after masking and particle detection in FIJI, defining thyroid follicle centres. (**b**) There was no difference in point intensity between normal and diseased (Hashimoto’s thyroiditis) thyroids but crude mean nearest neighbour distances were greater in point patterns from diseased thyroid. (**c**) Single value descriptors relating to point distribution, Clark and Evans Aggregation Index and Hopkins-Skellam index, were significantly different between groups (individual points and median (centre line), first and third quartiles (lower and upper box limits), 1.5 × interquartile range (whiskers); p-values of Welch unpaired two-sided two-sample t-test for each metric, n = 10).

To illustrate the quantification of features relationships that only this approach can provide in a user-independent manner, the fully-classified images of complete thyroid lobe transections were used in an open-source pipeline developed to take the annotation input from an image processing package through a specialised R package for spatial statistics. For convenience, the largest rectangular window common to all classified images was selected. The ‘colloid’ class, effectively identifying functional follicles, was separately masked, and (x,y) centroids of the individual follicles represented by this were used to generate spatial point patterns using the *spatstat* package within R (Fig. 5a).

The simplest measures of a spatial point pattern are point intensity and mean nearest-neighbour distance. The intensity of the point patterns in normal and diseased thyroid were not significantly different (Fig. 5b) but the mean nearest neighbour distance of the point pattern in diseased thyroids was significantly greater than in normal thyroids (p = 0.002128, Welch unpaired two-sided two-sample t-test, n = 10, Fig. 5c).

Single figure metrics derived from each point pattern can begin to evaluate the distribution of points (Fig. 5d). The Clark–Evans Aggregation Index is a simple measure of point clustering represented by the ratio of the mean nearest neighbour distance in a pattern to the mean distance in a pattern of complete spatial randomness (CSR) with the same intensity; a value < 1 suggests clustering and > 1 suggests ordering/dispersal. The mean value for both groups was > 1, suggesting follicle dispersal that was significantly greater in diseased thyroids (p = 0.000123, Welch unpaired two-sided two-sample t-test, n = 10). The Hopkins–Skellam Index also evaluates the nearest neighbour distances of a point pattern against those of a pattern of complete spatial randomness with the same intensity, where a value of 1 represents CSR, < 1 suggests point clustering and > 1 suggests dispersal. In contrast to the Clark-Evans Aggregation Index, the Hopkins-Skellam index value for normal thyroid suggested follicle clustering but follicle dispersal in diseased thyroids, with a significant difference between groups (p = 1.324e−05, Welch unpaired two-sided two-sample t-test, n = 10).

**Figure 6:**
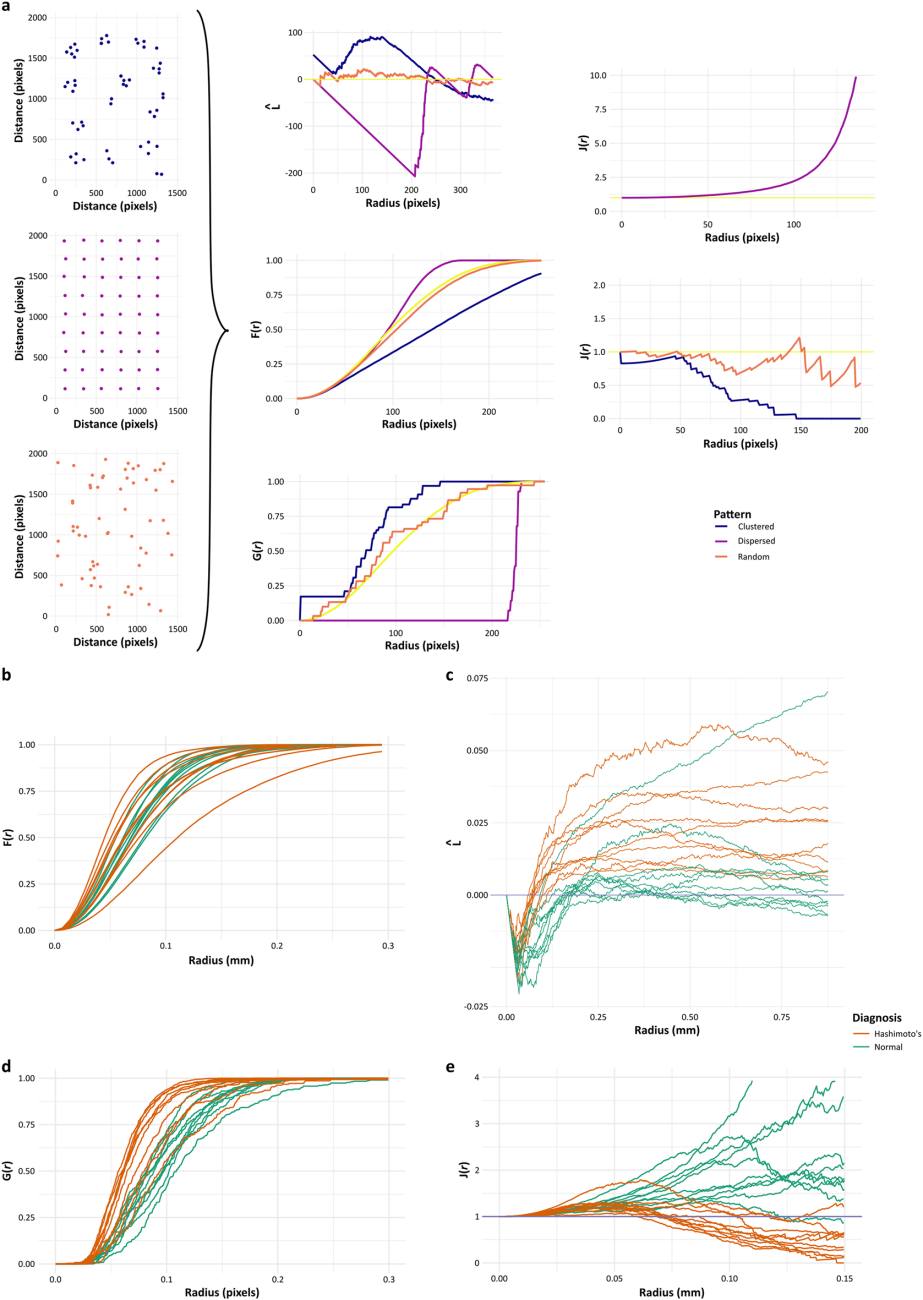
Specialised functions of spatial point patterns quantify disease-related histological features. (**a**) Ripley’s L-function and the F-, G- and J-functions are second moment properties of a spatial point pattern. Plots of these functions distinguish between point patterns showing clustering or dispersal compared with complete spatial randomness (CSR), and groupwise statistical comparisons of empirical functions can be made. Example plots of corrected Ripley’s L-function, F-, G- and J-functions using synthetic examples of clustering, dispersal and CSR are shown. (**b**) Individual empirical corrected Ripley’s L-function plots show greater clustering tendencies of follicles in Hashimoto’s thyroiditis (above the horizontal yellow line), compared with normal thyroid. (**c**) Individual empirical corrected F-function plots appear similar in normal and diseased thyroid although greater variation between cases is evident in diseased thyroid. (**d**) Individual empirical G-function plots also show greater clustering tendencies of follicles in Hashimoto’s thyroiditis. (**e**) The individual empirical summary J-function plots similarly show greater clustering tendencies of follicles in Hashimoto’s thyroiditis and suggest regular dispersal rather than CSR in normal thyroid.

Much information is lost by summarising point patterns by a single figure metric and more insightful functions for understanding and quantifying the relationship of points can be applied. In adjusted plots of the empirical Ripley’s L-function, CSR of features is represented by a horizontal line through zero on the y-axis; clustered features plot above the line of CSR, and feature regularity/dispersal is plotted below the line, indicated in the synthetic dataset plots (Fig. 6a). Other available functions utilise the space between points in addition to the points themselves. The empty-space function, F, is based on the distance from any point within the empty space to the nearest point. The nearest neighbour distance distribution function, G, provides greater information than the simple mean nearest neighbour distance, and the J-function is a summary function that incorporates both F and G functions. For each function, the plots of synthetic point patterns representing dispersal and clustering are shown (Fig. 6a).

The individual empirical F-function plots of follicle point patterns are similar for normal and diseased thyroids although with greater variation between cases in the diseased group, and the F-functions are not significantly different between groups [studentized permutation test for grouped point patterns, T (999 random permutations) = 0.33425, p-value = 0.196, Fig. 6b]. In contrast, the adjusted Ripley’s L-, G- and J-function plots show differences between groups, with plots indicating clustering of follicles in cases of Hashimoto’s thyroiditis, compared with plots consistent with randomness or dispersal of follicles seen in normal thyroid (Fig. 6c–e). The group-wise comparison indicates that each function is statistically different between groups (studentized permutation test for grouped point patterns, 999 random permutations: Ripley’s L-function, T = 7.3732, p-value = 0.005; G-function, T = 1.9981, p-value = 0.006; J-function, T = 2.4405, p-value = 0.001). Such differences can be qualitatively appreciated in the H&E images, where follicles in normal thyroid are largely evenly dispersed and those in Hashimoto’s thyroiditis are disrupted, often smaller, and with inflamed and fibrotic areas of follicular destruction that leads to apparent cluster formation. However, only analysis of spatial point pattern can quantify these subjective changes to architecture that are secondary to the inflammation that simpler methods evaluate.

### Spatial point pattern analysis of annotated features can identify and quantify occult deviation from microarchitectural normality

**Figure 7:**
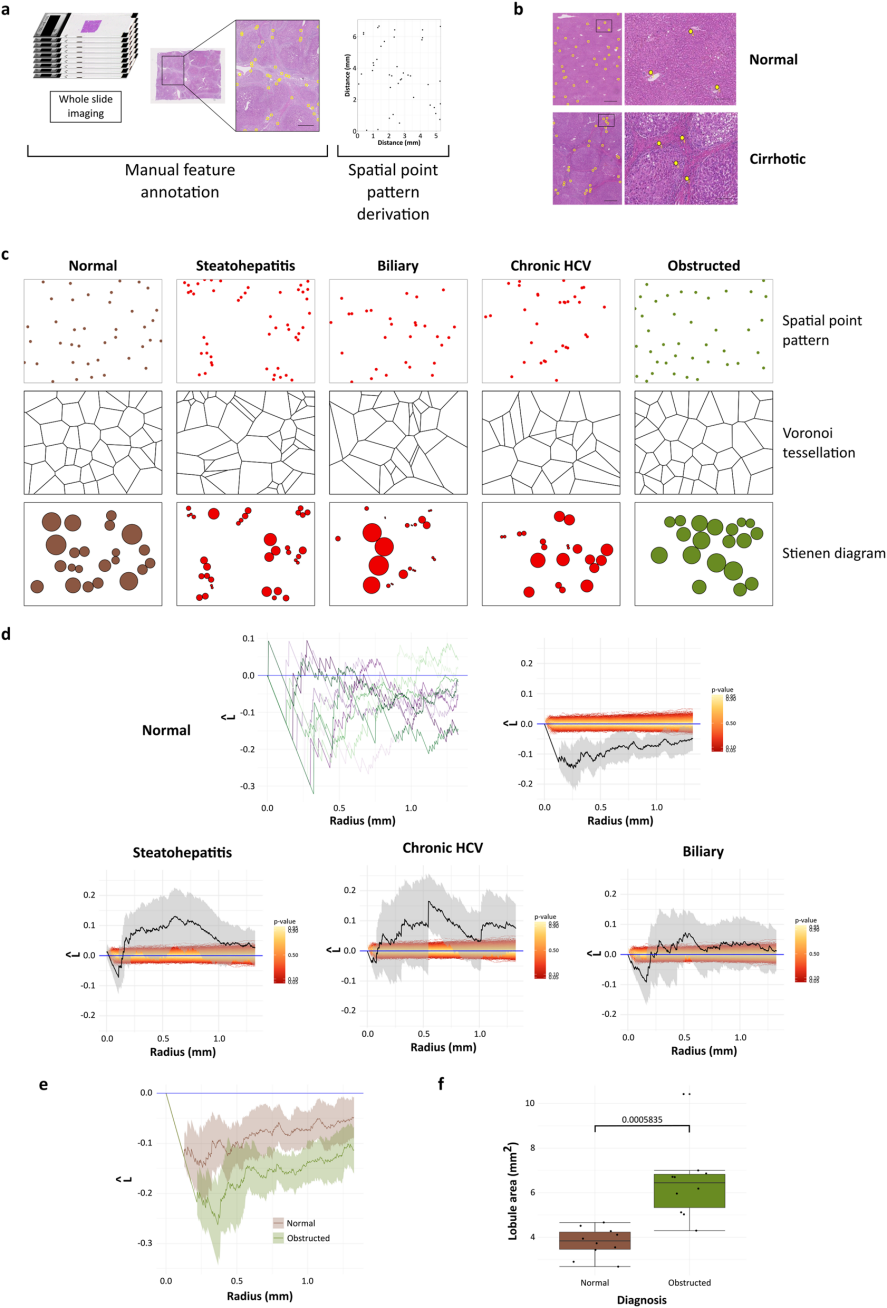
Spatial point pattern analysis of discrete features can confirm and quantify subjective ‘gold-standard’ evaluations and identify occult architectural abnormalities. (**a**) Specific features can also be manually annotated to generate spatial point patterns. Portal tracts in human liver were manually annotated in regions from whole-slide images by identification of hepatic artery branches. Scale bar 1 mm. (**b**) Example annotations of portal tracts in H&E-stained sections from explant cirrhotic livers and histologically normal liver. (**c**) Individual spatial point patterns of portal tracts in normal and cirrhotic liver of varying aetiology can be visualised as Voronoi tessellations or Stienen diagrams, indicating dispersal of portal tracts in normal liver and suggesting clustering in cirrhotic liver. (**d**) Generation of second moment property functions such as Ripley’s L-function allows quantification of these traditional and previously subjective central tenets of liver disease—loss of normal portal-central vascular relationships—by proving that significant portal tract regularity/dispersal in normal liver is lost in end-stage chronic liver disease where clustering tendencies are present (Ripley’s L-function with 95% confidence intervals, n = 10). Scale bars 1 mm. (**e**) Analysis of generated point patterns from peripheral liver in cases with hilar tumours demonstrated that portal tracts were significantly more dispersed in the livers from patients with hilar tumours (corrected Ripley’s L-function with 95% confidence intervals, n = 10). (**f**) Companion annotation of central vein profiles allowed portal-central distances to be calculated and demonstrated increased modelled lobular size (data represented as individual points with median (centre line), first and third quartiles (lower and upper box limits), 1.5 × interquartile range (whiskers), n = 10, p-value of Welch unpaired two-sample two-sided t-test).

Although computational annotation is convenient, manual annotation remains accurate for many applications. Targeted, high-fidelity, manual annotation of specific features permits hypothesis-driven interrogation using spatial point pattern landscape analysis, contrasting with the whole-landscape hypothesis-generating approach inherent to patch landscape analysis. Spatial point patterns were derived from manual annotations of large vascular structures in images of normal liver (n = 10), end-stage cirrhotic liver including cases showing the three dominant patterns of fibrosis (primary biliary disease (n = 11), steatohepatitis (n = 10), and chronic Hepatitis C virus infection as a cause of lobular hepatitis (n = 10), and peripheral liver from cases with central (hilar) tumours (cholangiocarcinomas, n = 10, Fig. 7a,b). An example Voronoi tessellation (where each tile for a given point of the point pattern represents the space in which every point within is closer to the given point than any other point of the point pattern) and Stienen diagram (where a circle is drawn around each point of diameter equal to the nearest-neighbour distance; circles outwith the window not plotted) from a spatial point pattern of normal liver and cirrhotic liver of each of three aetiological patterns allows an appreciation of the regularity and dispersal of portal tracts in normal liver and the tendency towards clustering seen in cirrhosis (Fig. 7c). Within the field of liver pathology, the loss of this regular hepatic architecture is the subjective histological *sine qua non* of end-stage liver disease. The empirical Ripley’s L function plot for portal tracts in normal liver demonstrates statistically significant regularity at all scales for each subject. Differences between aggregated Ripley’s L functions of each cirrhotic group and normal liver were statistically significant [Fig. 7d, studentized permutation test for grouped point patterns, Tbar (999 random permutations) = 8260.7, p-value = 0.011], formally quantifying this central tenet of liver pathology for the first time. This quantification offers support for the proposed mechanism of development of cirrhosis through parenchymal extinction that ‘draws together’ adjacent structures^15^. No alternative method exists for the quantification of subjective pathological feature disorganisation of this nature. Differences in Ripley’s L function plots between disease categories was evident, with more clustering apparent in chronic hepatitis C virus and steatohepatitis than in biliary disease, although these differences were not significant by aggregated comparison across the functions as a whole in these proof-of-principle cohorts.

The same approach was applied to peripheral liver from cases with central (hilar) tumours (cholangiocarcinoma), all clinically reported by specialist Hepatopathologists as having normal peripheral microarchitecture. Example Voronoi tessellation and Stienen diagram plots look qualitatively similar to those of normal liver (Fig. 7c). However, calculation of empirical Ripley’s L-functions demonstrated significant differences in the organisation of portal tracts in cases with hilar tumours compared with normal liver, with greater dispersal of portal tracts in the peripheral liver of cases with hilar tumours [Fig. 7e, studentized permutation test for grouped point patterns, Tbar (999 random permutations) = 1935.2, p-value = 0.006]. Additional annotation of central veins allowed calculation of inter-vascular distances by calculating the nearest-neighbour distances between points of different classes, permitting the size of liver lobules, a microarchitectural functional unit, to be modelled based on the two-dimensional lobule-as-hexagon paradigm. This confirmed statistically significant pathological lobular enlargement (Fig. 7f, p = 0.0005835, Welch unpaired two-sample two-sided t-test, n = 10). Thus, targeted complementary spatial point pattern metrics readily defined both previously unquantifiable subjective features and occult disease-related structural changes that were not apparent by specialist gold-standard subjective assessment.

Image sets from obstructed and normal renal cortex (Supplementary Fig. 1), and normal pancreas (Supplementary Fig. 2), were also examined to demonstrate additional multi-organ applicability. Glomerular and Islet of Langerhans distributions, respectively, could be quantified by the same functions. The renal cortex in centrally obstructed kidneys did not demonstrate derangement of normal architecture equivalent to that found in the liver, in keeping with the fundamental differences in organ plasticity and responses to injury.

### Individual cell annotation allows quantification of fine-grain cellular relationships that generates new insights into fundamental disease processes

**Figure 8:**
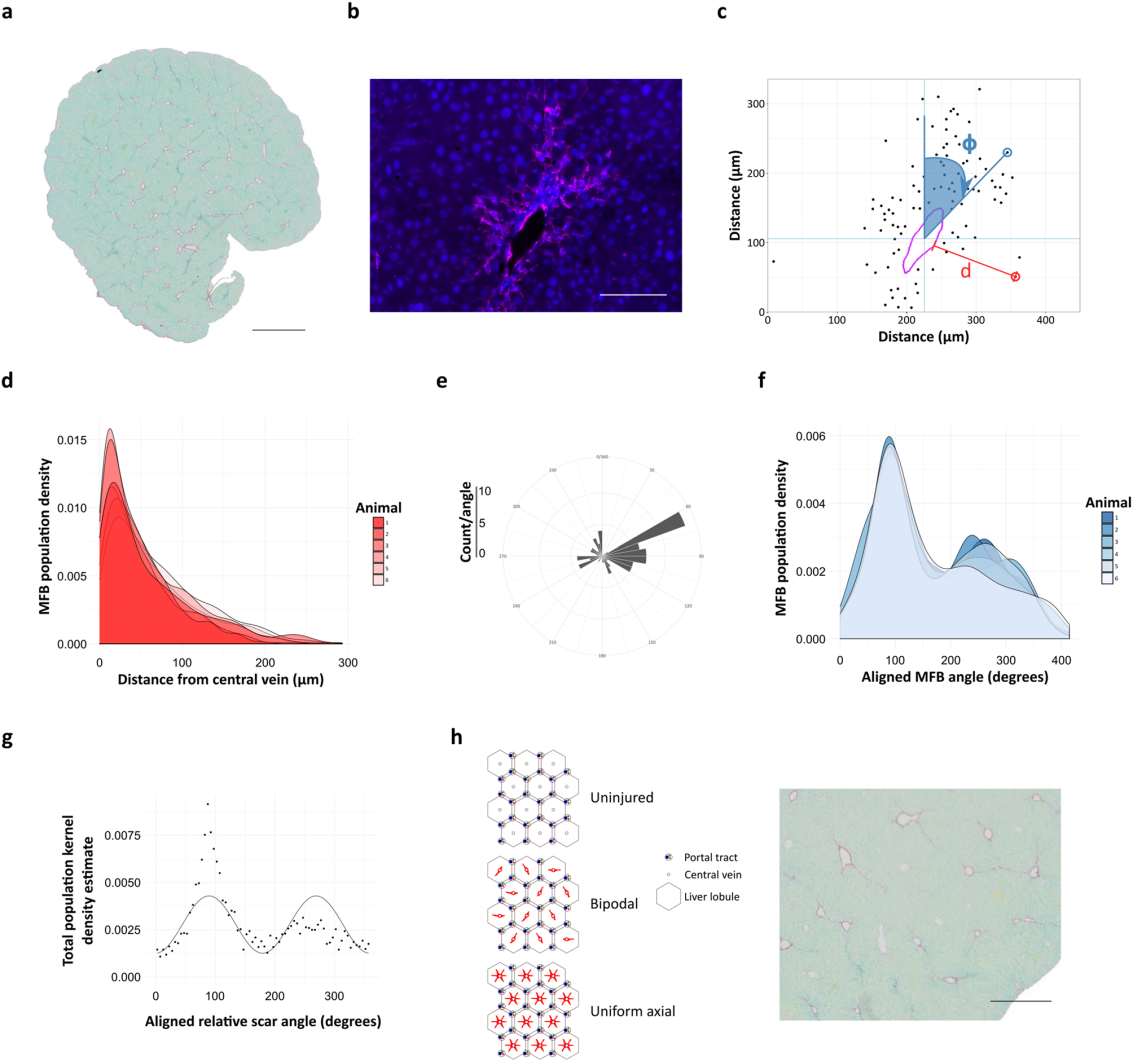
Individual cell annotation allows quantification of fine-grain cellular relationships that derives new insights into fundamental processes in translational models of disease. (**a**) Early pericentral hepatic fibrosis (picro-sirius red-stained, scale bar 1 mm), was induced in a cohort of wild type mice (n = 6), and sections stained for αSMA to identify MFBs (**b**, lilac, scale bar 100 μm). (**c**) 10 pericentral fields from each were used to annotate the nuclear position of each MFB, and the circumference of the central vein, to generate spatial point patterns from which the distances (**d**, red) of individual cell from vessels (lilac) and relative polar angle of individual cells with respect to vessel lumen centroids (ϕ, blue) could be calculated. (**d**) Scar phenotyping by density distribution of calculated MFB-central vein distances (**d**) for each animal demonstrates an MFB gradient within scars, highest at the central veins. (**e**) The nuclear position of each MFB was converted to polar coordinates with reference to the calculated centroid of the annotated vessel. The polar angle of the peak of the kernel density estimate of all polar MFB angles was set to 90° by ‘rotating’ all MFBs about the central vein centroid to allow alignment of all images. (**f**) Aggregates of aligned MFBs plotted for each animal as a polar histogram or kernel density estimate demonstrated a dominant pericentral spur with a smaller secondary antipodal spur. (**g**) The data can be fitted by a sine wave with 180° equivalent periodicity. (**h**) Diagrammatic representation of normal liver lobules with the classic hexagonal arrangement, and diffuse pericentral fibrosis or with dominant and single antipodal spurs; visual comparison of identical murine liver stained with picro-sirius red shows that liver scarring is organised with a dominant scarring axis, accompanied by a single secondary directly-opposed axis rather than developing uniformly along all available central-central axes (scale bar 500 μm).

The relational context of cells, as well as tertiary structures, can also be defined by a spatial point approach to generate additional mechanistic insight. A dataset of images from a rodent model of early scarring in fatty liver disease in which scar-orchestrating (α-smooth muscle active-positive) myofibroblasts (MFBs)^16^ had been immunofluorescently stained (Fig. 8a) was used for manual annotation of both the positions of MFB nuclei and the focal point of injury in this model, the central veins (Fig. 8b). Separate spatial point patterns of MFBs and the central vein circumference were used to define the relative MFB positions with reference to the central vein profile, providing information about individual cell distance and orientation (Fig. 8c). The scar axes could be determined by calculation of the radial MFB densities, and alignment of the calculated dominant axis in each field allowed all fields to be compared.

The distribution of MFB-to-central vein distances (Fig. 8d) can provide quantitative phenotypic histological data beyond crude cell number^17^. Relative scar axis based on peak MFB density (Fig. 8e,f) was determined for each animal, and examination of this fundamental disease process in relation to a fixed histological landmark revealed that scarring is initiated in a bipolar manner (Fig. 8g), rather than along all possible axes, indicating an unknown property of scar initiation (Fig. 8h) that can be subjectively appreciated in low-power images.

## Discussion

Our framework depends upon a simple conceptual shift to consider a histological section as a tissue landscape, releasing the rich topography for interrogation by the methodologies of geosciences and landscape ecology. Classifier-agnostic hypothesis-generating whole landscape analysis can be undertaken using patch landscape ecology tools. The user-specified suite of metrics describes previously unquantifiable feature relationships over all microarchitectural scales. Critically, given the proliferation of computational methods to quantify images, this approach to a fully segmented classified image permits a complete suite of new metrics to be generated in a species-, tissue-, disease-, or segmentation methodology-agnostic manner without any additional training requirement.

In contrast, computationally derived or targeted manual feature annotation allows spatial point pattern analysis and phenotyping, a complementary framework for interrogating the histological landscape. Features only previously subjectively assessable can be quantified to phenotype and describe normal and diseased histological landscapes and derive mechanistic insight.

Exemplar applications using liver and thyroid disease encompassing inflammatory, fibrotic and neoplastic pathology are presented but the framework can be applied to any tissue image set once histological fluency has informed the specific research question.

## Methods

### Human tissue access

Human tissue was obtained by approved application to the Lothian NRS Human Annotated Bioresource that is authorised to provide unconsented anonymised tissue under ethical approval number 15/ES/0094 from the East of Scotland Research Ethics Service REC 1. All tissue was from cases from 2006 onwards and received anonymised to all details other than aetiology.

For manual annotation studies, single haematoxylin and eosin-stained sections from the deep hepatic parenchyma, sampled as part of the standard diagnostic specimen pathway, were used. No additional sections were required. Sections were obtained from cirrhotic explants with the 3 dominant patterns of fibrosis; primary biliary disease (n = 11), steatohepatitis (n = 10), and chronic Hepatitis C virus infection as a cause of lobular hepatitis (n = 10). 10 non-lesional deep parenchymal blocks (> 5 cm from hilar lesional tissue) from liver with hilar cholangiocarcinoma were also obtained. 10 non-lesional liver sections from partial hepatectomies for metastatic disease (eight colorectal carcinomas, one melanoma) or a benign biliary cyst (single case) were used to represent normal liver.

8 cases of non-lesional pancreas from pancreaticoduodenectomies (Whipple’s procedure) for extrahepatic cholangiocarcinoma arising proximal to the confluence with the pancreatic duct were used to represent normal pancreas.

Routinely sampled blocks of non-lesional renal cortex from nephrectomies from ten cases of conventional clear cell renal cell carcinoma, representing normal renal cortex and analogous to non-lesional blocks from partial hepatectomies for intrahepatic mass lesions, and from ten cases ureteric or renal pyloric urothelial carcinoma, analogous to the hilar cholangiocarcinomas, were used.

For automated segmentation, single haematoxylin and eosin-stained sections including lesional (hepatocellular carcinoma) and adjacent non-lesional liver were obtained from 54 explants or resections containing hepatocellular carcinoma, without selection for aetiology or tumour grade.

### Tissue image library access

Whole-slide images of PAXgene fixed, paraffin-embedded H&E sections of thyroid from autopsies in .svs format were downloaded from the GTEx Tissue Image Library. 10 cases documented as ‘Normal and 10 as ‘Hashimoto’s thyroiditis’ in the ‘Pathology Review Comments’ field. Autolysis for each was graded as ‘0’ or ‘1’.

### Murine model of liver fibrosis

Liver fibrosis was induced in cohorts of wild type C57Bl6 male mice by 8 weeks carbon tetrachloride (CCl_4_) injection twice weekly, 0.25 µl/g body weight in a 1:3 ratio with sterile olive oil^18^ or vehicle alone. Animals were not randomised to injury or control groups. Blinding to control or injury groups was not possible as injury is macroscopically and microscopically apparent. Animals were housed in a specific pathogen-free environment and kept under standard conditions with a 12 h day/night cycle and access to food and water ad libitum. All animal experiments were carried out under procedural guidelines, severity protocols and with ethical approval from the University of Edinburgh Animal Welfare and Ethical Review Body and the Home Office (UK).

### Scanning and image generation methods

Whole slide images of haematoxylin and eosin-stained human sections in .ndpi format were acquired using a Hamamatsu NanoZoomer to × 20 depth. Tiled-TIFF thumbnails were generated from the .ndpi files using ndpisplit from the NDPITools suite^19^, and tiled-TIFF files converted to standard TIFF (for automated segmentation) or JPEG (for manual annotation) format compatible with ImageJ^20^ by command-line ImageMagick.

### Immunofluorescence methods

Antigen retrieval of murine sections was achieved by microwaving in Tris–EDTA pH 9.0 for 15 min.

For immunofluorescent staining of aSMA, sections of murine liver were labelled with a monoclonal mouse antibody (Sigma A2547, clone 1A4, 1:1500 dilution, 1-h incubation at room temperature). Staining was visualized with donkey anti-mouse IgG (H and L) Alexa Fluor 555 conjugated secondary antibody (ThermoFisher Scientific), and sections mounted in VECTASHIELD HardSet Antifade Mounting Medium with DAPI (Vector Laboratories). Negative controls were performed using identical concentrations of species and isotype-matched non-immune immunoglobulin in place of primary antibody or omission of primary antibody.

10 × 20 objective fields centred on a central vein (in keeping with the pattern of damage of CCl_4_) were acquired using a Zeiss Axioplan II microscope and Photometrics CoolSNAP HQ2 camera, and separate TIFF images of each channel exported.

### Manual identification and annotation of histological features

For human liver tissue, a central 5.32 mm × 7.11 mm (37.8 mm^2^) rectangular field from each .jpeg thumbnail whole slide image, the largest that could be taken from every scan, was cropped in FIJI^12^ and used to mark, as separate region of interest (ROI) sets, the centre of each central vein (from normal or centrally obstructed) and centre of each hepatic artery (identifying portal tracts when paired with a portal vein branch and/or bile duct). Marking was informed by viewing the WSIs in NDPIviewer (Hamamatsu) alongside to allow accurate identification.

For human pancreatic tissue, a central 5.32 mm × 7.11 mm (37.8 mm^2^) rectangular field from each .jpeg thumbnail whole slide image was used to mark the centre of each islet of Langerhans.

For human kidney, a 4.54 mm × 2.72 mm (12.35 mm^2^) rectangular field of renal cortex from each .jpeg thumbnail whole slide image was used to mark the centre of each glomerulus.

For murine myofibroblast (MFB) images, multichannel images were created in FIJI using the Image5D plugin, and the nucleus of each aSMA-positive MFB marked manually as an ROI set, excluding nuclei of concentrically arranged smooth muscle cells in vessel walls. The circumference of the central vein lumen also marked as a separate line segment ROI.

### Computational image segmentation

#### Liver classification in FIJI

1 mm^2^ ROIs from lesional (HCC) and non-lesional liver from each resection or explant case were selected manually and used to create 4 contiguous tiles from each.

A WEKA machine-learning classifier was trained in FIJI by a specialist liver transplant pathologist at the national liver transplant centre to simply deconvolve the staining into haematoxylin (nuclei), eosin (cytoplasm) and unstained areas (sinusoids/vessels). The classifier was applied to all tiles using a script that generated a classified TIFF output image.

#### Thyroid classification in QuPath

Downloaded .svs files were opened in FIJI using the Bio-Formats plugin, and ‘Series 4’ of the container format extracted and converted to an RGB composite image. The image was cropped to a single full transection of thyroid and saved in .tiff format.

A pixel classifier was trained in QuPath 0.2.2 using RTrees with ‘gaussian’ and ‘weighted deviation’ features selected at ‘Very high’ (0.49 μm/pixel) resolution^21^. Pixels were classified as one of ‘cells’, ‘stroma’, ‘colloid’, and ‘space’ and a categorical classified .tiff saved as an output. The number of pixels of each class within a separate ‘all_tissue’ mask was also generated.

### Thyroid follicle point pattern generation

Each classified .tiff thyroid image was cropped to a size of 1773 × 1850 pixels, including tissue only. A script to select the ‘colloid’ class, convert to mask, fill holes, and outline rounded structures and generate to structure centroids using the ‘Analyze particle’ tools was run. Outlined images and centroids in .csv format were generated.

### Spatial point pattern analysis

Spatial point pattern and statistical analysis were undertaken in the RStudio^22^ environment for R. For each liver image, FIJI generated ROIs were imported using the *RImageJROI package*^23^ read.ijroi() function, and converted into *spatstat* package^24^ spatial point patterns using the ij2spatstat() function. For thyroid follicle centroids, spatial point patterns were created directly from imported .csv files.

Spatial point pattern analysis was performed using the *spatstat* package. For distribution analysis of tertiary and quaternary structures in human tissue (portal tracts, central veins, islets of Langerhans, glomeruli, thyroid follicles), Ripley’s L-function^14^ was implemented with the Lest() function with the default edge corrections (Ripley’s isotropic, translation and border) applied; global envelopes using Monte-Carlo simulations of the theoretical L-function of complete spatial randomness (CSR) were derived by the envelope() function. All other spatial point plots, metrics and functions were generated using appropriate *spatstat* functions with defaults. Empirical functions L, F, G, and J of groups were compared with the studpermu.test() function.

To estimate individual lobule size based on the classical lobule depiction as a regular hexagon in normal and obstructed human liver, the distances from each central vein to the 6 nearest portal tracts were calculated with the nndist() function. For each central vein, the mean to the 6 distances (r) was used to calculate the area of the lobule $\left( {\frac{{3\sqrt {\left( 3 \right)} }}{2} \times r^{2} } \right)$.

For analysis of central vein-MFB radial distances, the nncross() function was used to determine the shortest distance to the central vein circumference for each aSMA-positive cell nucleus. For MFB directional analysis, the centroid of the central vein for each image was calculated using the centroid.owin() function, and used as (0,0). The position of each MFB was converted to polar coordinates to calculate the angle (ϕ_i_) from an arbitrary reference. Kernel density estimation of all MFB ϕ_i_ for each image was calculated with the density() function of the core stats package, and the angle of peak density (ϕ_peak_) determined. To allow comparison with distributions of MFBs from other images, all MFBs were effectively rotated about the central vein centroid such that ϕ_peak_ was 90°.

### Patch-based landscape analytics

Classified TIFF output images from WEKA/FIJI or QuPath were used in a pipeline in RStudio that first converted each to a GeoTIFF image using the Universal Transverse Mercator projection and World Geodetic System (WGS) 84 datum using *rgdal* package accessing the Geospatial Data Abstraction Library^25^ and PROJ.4^26^. GeoTIFF images were used as input for the *landscapemetrics* package^27^ to analyse the categorical landscape patterns using metrics based on the FRAGSTATS suite^9^ as well as more recently developed measures of landscape complexity^10^. Heatmaps were generated with the *ComplexHeatmap* R package, with k = 2 k-means clustering of cases^28^.

### Machine learning disease classification

The paired HCC and non-lesional classified image set was used. Eighty per cent of cases were randomly chosen as a training set and the remainder used only as a validation set.

Landscape and class level metrics of the ‘aggregation’, ‘area and edge’, ‘diversity’, and ‘complexity’ groups were used as features for model training after near-zero variance features were removed using *caret::nearZeroVar*^29^. Features of the training set were optimally normalised using *bestNormalize*^30^, and features selected for model training by removal of those that were highly correlated (> 0.75). A random forest model with 10,000 trees was constructed to predict disease classification (HCC or non-lesional) using *randomForest*^31^. Variable importance measures of the constructed forest^32,33^ were calculated using *randomForestExplainer*^34^.

### Third-party geographical images

A satellite image from the European Space Agency Copernicus Sentinel-2B satellite L1C 2019-02-26 dataset was retrieved using the Sentinel Hub EO Browser under CC BY 4.0. The corresponding mapped region was retrieved from OpenStreetMap under Open Database License (Copyright OpenStreetMap contributors) to generate the composite image.

### Statistical methods

Distributions of MFB subpopulations were evaluated with a bootstrap version of the Kolmogorov–Smirnov test, ks.boot(), in the *Matching* package^35^.

For inter-group comparison of lobular area and central vein-MFB distances, normality of data was determined using Shapiro–Wilk testing and by examination of qq plots. After assumptions of normality were satisfied, the Welch (unequal variance) t-test was used to compare two groups^36^.

## Supplementary information

Supplementary file1

Supplementary file2

## Data Availability

Raw images are available on reasonable request. Scripts for patch landscape generation and spatial point pattern analysis in R, classification and pixel quantification in QuPath, and centroid determination in FIJI are available from https://github.com/TKPath/landscape_histology.
