# Supplementary material for: Integration of geoscience frameworks into digital pathology analysis permits quantification of microarchitectural relationships in histological landscapes
Source: Sci Rep. 2020 Oct 16;10:17572. doi: 10.1038/s41598-020-74691-9 (PMC7567886; doi:10.1038/s41598-020-74691-9)
Supplement: Supplementary file 1 — Supplementary file1 [file 41598_2020_74691_MOESM1_ESM.pdf]

Integration of geoscience frameworks into digital pathology analysis permits quantification of microarchitectural relationships in histological landscapes

Timothy J Kendall, Catherine M Duff, Andrew M Thomson, John P Iredale

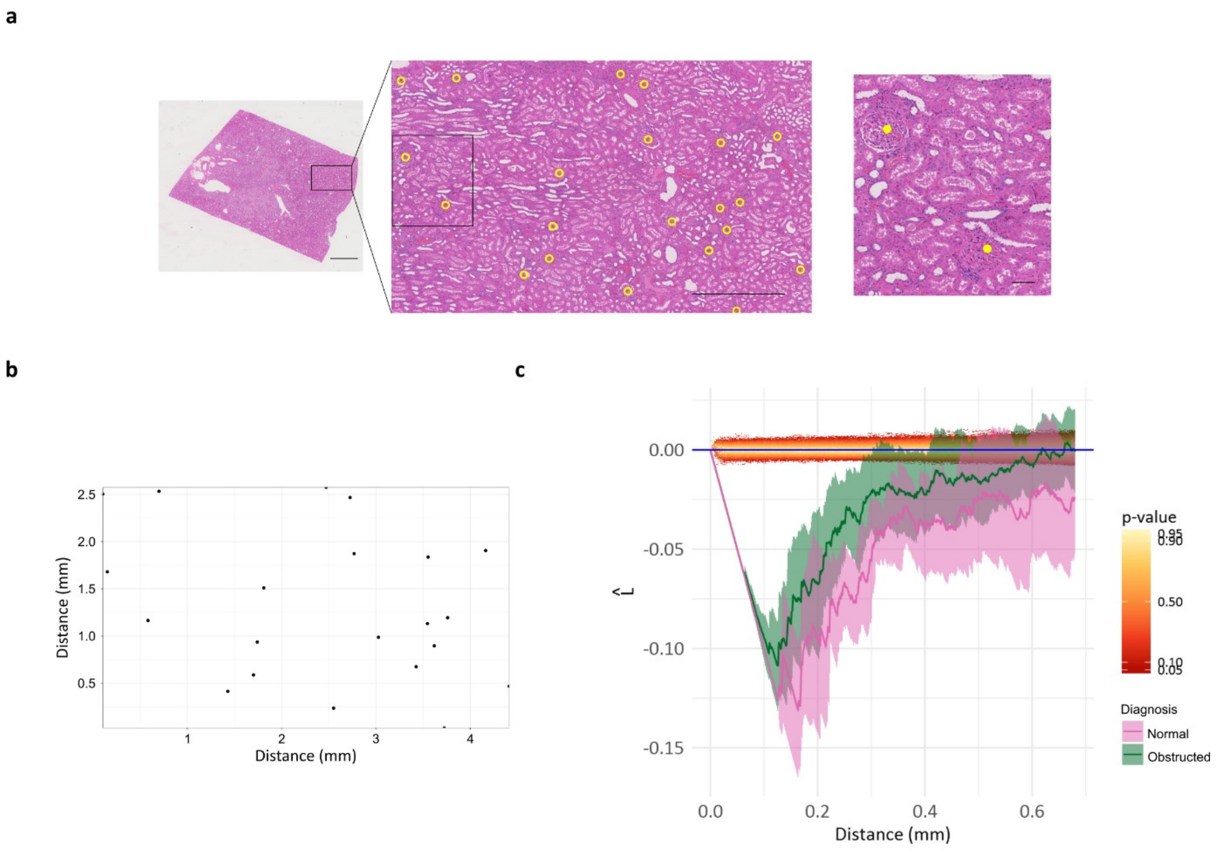

Supplementary Figure 1. Renal cortical landscape assessment by spatial point pattern analysis. A. Fields from the cortex of normal kidney and from the kidneys with tumours of the renal pelvis were annotated to mark the glomeruli. Scale bars 3 mm left, 1 mm centre, 100 µm right. B. Glomerular positions were used to create spatial point patterns. C. Groupwise comparisons of Ripley's L-function demonstrate glomerular dispersal that is unchanged in obstructed organs (Ripley's L-function with 95% confidence intervals, n=8).

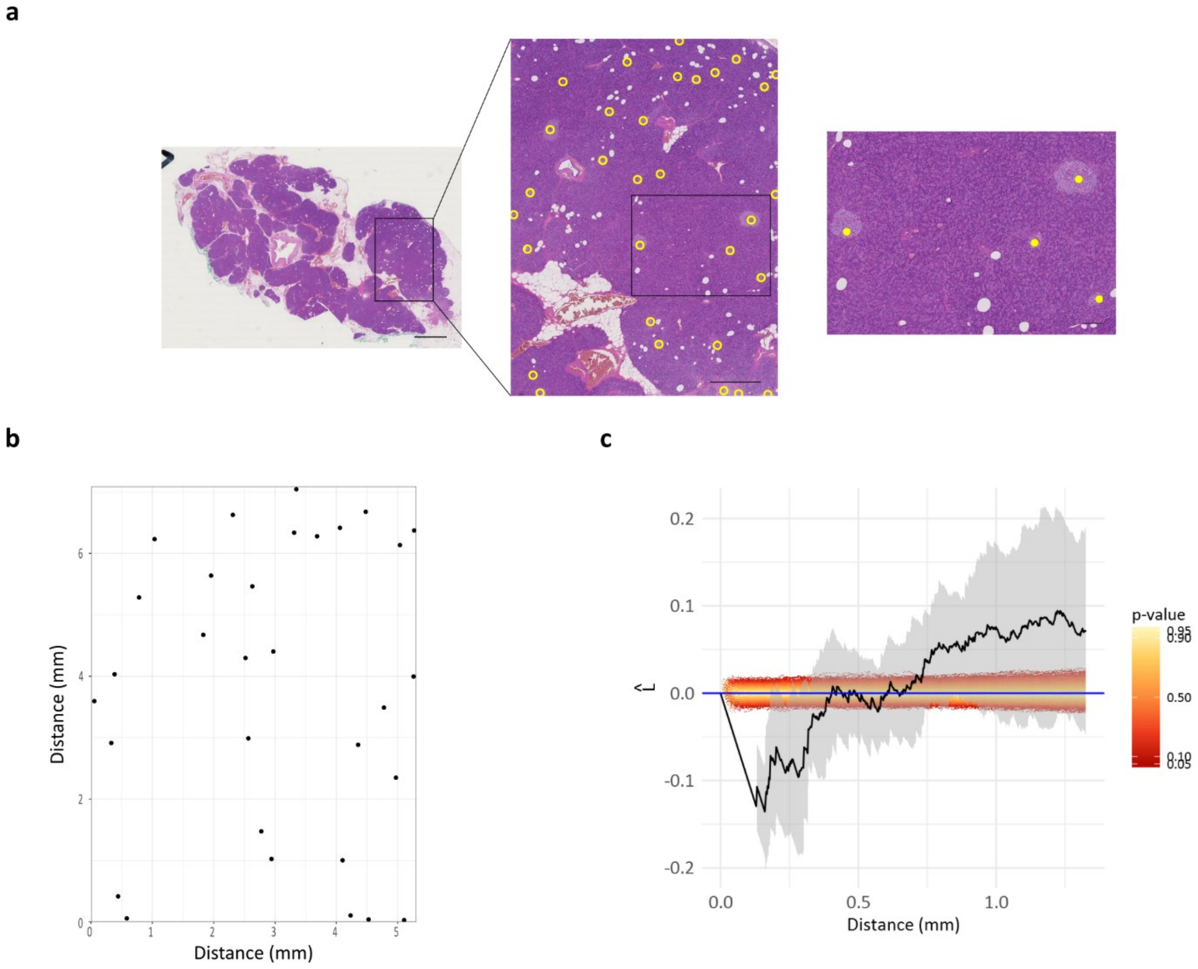

Supplementary Figure 2. Pancreatic landscape assessment by spatial point patterns. A. Islets of Langerhans were annotated in images of normal pancreas and used to create spatial point patterns (B). Scale bars 3 mm left, 1 mm centre, 200 µm right C. The distribution of islets throughout the pancreas was not statistically separable from complete spatial randomness by Ripley's L-function evaluation, n=10.
